# Predictive Role of Urinary Metabolic Profile for Abnormal MRI Score in Preterm Neonates

**DOI:** 10.1155/2018/4938194

**Published:** 2018-10-01

**Authors:** Maria Luisa Tataranno, Serafina Perrone, Mariangela Longini, Caterina Coviello, Maria Tassini, Antonio Vivi, Marco Calderisi, Linda S. deVries, Floris Groenendaal, Giuseppe Buonocore, Manon J. N. L. Benders

**Affiliations:** ^1^Department of Neonatology, Brain Center Rudolf Magnus, University Medical Center Utrecht, Utrecht University, Utrecht, Netherlands; ^2^Department of Molecular and Developmental Medicine, University of Siena, Siena, Italy; ^3^Division of Neonatology, Careggi University Hospital of Florence, Florence, Italy; ^4^NMR Centre, University of Siena, Siena, Italy; ^5^Kode srl, Pisa, Italy

## Abstract

**Background and Objective:**

Early identification of neonates at risk for brain injury is important to start appropriate intervention. Urinary metabolomics is a source of potential, noninvasive biomarkers of brain disease. We studied the urinary metabolic profile at 2 and 10 days in preterm neonates with normal/mild and moderate/severe MRI abnormalities at term equivalent age.

**Methods:**

Urine samples were collected at two and 10 days after birth in 30 extremely preterm infants and analyzed using proton magnetic resonance spectroscopy. A 3 T MRI was performed at term equivalent age, and images were scored for white matter (WM), cortical grey matter (cGM), deep GM, and cerebellar abnormalities. Infants were divided in two groups: normal/mild and moderately/severely abnormal MRI scores.

**Results:**

No significant clustering was seen between normal/mild and moderate/severe MRI scores for all regions at both time points. The ROC curves distinguished neonates at 2 and 10 days who later developed a markedly less mature cGM score from the others (2 d: area under the curve (AUC) = 0.72, specificity (SP) = 65%, sensitivity (SE) = 75% and 10 d: AUC = 0.80, SP = 78%, SE = 80%) and a moderately to severely abnormal WM score (2 d: AUC = 0.71, specificity (SP) = 80%, sensitivity (SE) = 72% and 10 d: AUC = 0.69, SP = 64%, SE = 89%).

**Conclusions:**

Early urinary spectra of preterm infants were able to discriminate metabolic profiles in patients with moderately/severely abnormal cGM and WM scores at term equivalent age. Urine spectra are promising for early identification of neonates at risk of brain damage and allow understanding of the pathogenesis of altered brain development.

## 1. Introduction

In developed countries, approximately 1.5% of neonates are born extremely preterm and despite the high rate of survival, the percentage of severe or moderate neurodevelopmental disability remains high, depending on gestational age and neonatal morbidity [1, 2]. Thus, early identification of neonates at risk for brain injury is of outmost importance to start appropriate intervention, but despite the large number of investigations aiming to early identify high risk infants, prediction of the long-term outcome is still challenging. Magnetic resonance imaging (MRI) is an additional tool to estimate the wide spectrum of preterm brain injury and, when performed at term equivalent age (TEA), is able to predict the adverse neurodevelopmental outcome [3]. In particular, white matter (WM), cortical grey matter (cGM), and cerebellum are the largest and most vulnerable brain structures in extremely preterm neonates [4]. Among all, cerebral WM is the most commonly involved site of injury in preterm infants [5]. During the last years, many attempts were made to define a comprehensive and objective MRI scoring system in order to describe the severity of injury and to correlate it with the long-term outcome. In 2013, Kidokoro and colleagues developed an MRI scoring system, providing a reliable brain injury classification that can be used at term equivalent age (TEA) [6]. Together with MRI, the use of biomarkers might be useful to predict, diagnose, and monitor brain injury and development. In this context, a new promising tool for disease prediction is proton magnetic resonance spectroscopy (^1^H-NMR) in biological fluids and, particularly, in urine [7]. Urinary metabolomics can be a potential, noninvasive biomarkers of brain disease. Urine, differently from blood, does not undergo homeostatic mechanisms and may reflect the body status, being informative for some diseases [7, 8]. Urinary metabolomics was found to be a good diagnostic tool, with high sensitivity and specificity for psychiatric disorders such as major depression, bipolar disorder, and autism spectrum disorders [9–11]. Furthermore, some studies showed the possible use of urinary metabolomics in the early diagnosis of cerebrovascular diseases, such as stroke [12] and neurodegenerative diseases (Alzheimer's and Parkinson's diseases, multiple sclerosis, and transmissible spongiform encephalopathy) [13–15]. Interestingly, a study by Ottens and colleagues demonstrated that urinary metabolomics was reliable in identifying patients with traumatic brain injury and in monitoring the evolution of rehabilitation [16].

Our aim was to study the urinary metabolic profile at 2 and 10 days after birth in preterm neonates with and without a severely abnormal MRI score at TEA.

## 2. Materials and Methods

### 2.1. Population

Sixty-five extremely preterm neonates with a gestational age (GA) <28 wks consecutively born from July 2012 till June 2013 at the Wilhelmina Children's Hospital in Utrecht were eligible for this prospective single center study. Infants were excluded if they had congenital malformations or if there was a clinical suspicion of genetic or metabolic disorders. The study was approved by the hospital medical ethics committee. Clinical investigations were conducted according to the principles expressed in the Declaration of Helsinki. All infants were scanned around TEA according to the clinical protocol. Written informed consent was asked to the parents of all the eligible infants, and twenty-five neonates were excluded since parents did not give consent for the enrollment. Eight neonates died in the neonatal period, thus were excluded from the study since MRI at TEA was not available. One infant did not have available MRI at TEA, and one more infant showed signs of a metabolic disorder; thus, they were both excluded from the study. A total of thirty infants were included in final analysis. Furthermore, clinical parameters and data on the occurrence of prematurity-related diseases such as intraventricular hemorrhage (IVH), necrotizing enterocolitis (NEC), patent ductus arteriosus (PDA), and bronchopulmonary dysplasia (BPD) and other clinical data were collected from patients' charts. All infants received the same parenteral nutrition, and the daily steps in oral feeding increase were done, following the local protocol for enteral and parenteral nutrition of the Wilhelmina Children's Hospital/UMC Utrecht, depending on the tolerance of the neonate. Mother milk or formula milk, when mother milk was not available, was used for enteral nutrition. Parenteral nutrition was introduced on day one after birth with a solution of glucose and amino acids (Primene 10%). Lipids (Intralipid 20%) were added between day one and two after birth. Protein and lipid intakes were progressively increased to a maximum of 3 g/kg proteins on day three to four after birth and 2.5 g/kg lipids between day four and five after birth.

### 2.2. Urine Samples and Storage

Urine samples were collected at 2 (between 44 and 50 hours) and 10 (between 238 and 242 hours) days after birth. Samples were noninvasively collected by the nurses, using a small plastic bag from which 2 ml of urine was withdrawn. Each sample was assigned with a patient anonymized number and sampling time. Samples were frozen at −80°C soon after sampling and stored at the Wilhelmina Children's Hospital/UMC in Utrecht. All urine samples were shipped in dry ice to the Nuclear Magnetic Resonance (^1^H-NMR) Laboratory of the University of Siena for the ^1^H-NMR analysis.

### 2.3. ^1^H-NMR Analysis

Urine NMR measurements were performed on a Bruker DRX 600 MHz Avance Spectrometer with a selective inverse probe (SEI) equipped with Z gradient coil. Spectra were acquired at a constant temperature of 298.0 ± 0.1 K by using a 90° pulses. Furthermore, 10 seconds delay was included in the pulse sequence to allow T1 relaxation. In fact, T1 values (in the range 1.5–2.8 s) of the analyzed metabolites are such that a 10 s delay allows full recovery of longitudinal magnetization after a 90° pulse, as verified by constant integral values for D1 ≥ 5 s. A 0.3 Hz line broadening function was applied before Fourier transformation. A saturation pulse of 2 s duration was applied at the water resonance to suppress the water signal. 32 K data points per scan were used, and 128 transients were accumulated. Each urine sample was first centrifuged at 2000 rpm for 5 min and analyzed afterwards. Sample (550 *μ*l) plus 50 *μ*l of a TSP-d4 20 mM solution were measured into a 0.5 mm (outer diameter) MR tube. All spectra were first run at their own physiological pH; we use this first spectrum only for an overview of the contained metabolites; then, we adjust the pH at 2.50 ± 0.02 in the same MR tube, with a microelectrode, and we run a second spectrum. The chemical shift of ionizable fluids is highly dependent on the pH. At a pH of 2.50, all chemical shift values are reproducible within ±0.01 ppm [17]. Moreover, under the described conditions, the methyl signals of creatine and creatinine are clearly separated (3.05 ppm for the methyl signal of creatine and 3.13 ppm for creatinine) and the methyl signal of lactic acid (1.41 ppm) is not overlapped by the methyl resonance of threonine (1.33 ppm). The pH was adjusted using a minimal volume of HCl, starting from a 3 M and ending with a 0.05 M, and samples were directly frozen at −80°C. All samples were run at the same time.

### 2.4. MRI Acquisition

The brain MRI was performed on a 3 T MR system (Achieva, Philips Medical Systems, Best, Netherlands) at TEA. Infants were positioned within a vacuum pillow in a SENSE head coil. The protocol included T2-weighted imaging in the coronal plane (repetition time 4847–6293 ms; echo time 120–150 ms; slice thickness 1.2 mm; in-plane spatial resolution 0.35 × 0.35 mm^2^, full brain coverage) and coronal T1-weighted imaging (repetition time 9.5 ms; echo time 4.6 ms; slice thickness 1.2 mm, full brain coverage). An experienced neonatologist was present during the examination. Oxygen saturation, respiratory rate, and heart rate of the infants were continuously monitored. If necessary, infants were sedated using oral chloral hydrate using a dose of 50–60 mg/kg through the gastric tube. All infants received double-layer hearing protection using MiniMuffs (Natus Medical Incorporated, San Carlos, CA, USA) and earmuffs (EM's 4 Kids, Brisbane, Australia).

### 2.5. MRI Scoring System

All MRI images were scored for the presence of brain abnormalities by two experienced neonatologists (LdV and MB) with more than 20 years' experience in neonatal neuroimaging, using the Kidokoro scoring system [6]. According to the scoring system, white matter (WM), cortical grey matter (cGM), deep GM (dGM), and cerebellar abnormalities were evaluated. The score class for each region (WM, cGM, DGM, and cerebellum) was calculated, obtaining the following classes: normal, mildly abnormal, and moderately and severely abnormal classes, according to Kidokoro et al. [6]. Scorings were performed using OsiriX (32-bit version, http://www.osirix-viewer.com), which allowed conversion to all planes.

### 2.6. Statistical Analysis

All statistical analyses were carried out on preprocessed data. Unit area normalization, Pareto scaling, and mean centering have been performed on each spectrum before analysis. In order to analyze the differences in the metabolic profile that are connected to the different newborn status, we used the two-step analytical procedure (often used in metabolomics): first, we use an unsupervised technique, principal component analysis (PCA), to find trajectories and clustering, and second, we model the system by using a classification technique, in this case partial least square discriminant analysis (PLSDA). Finally, using PLSDA, the discriminant performance of spectra has been checked and receiver-operating characteristic (ROC) curves were obtained, in order to verify the ability to distinguish infants with a later moderately/severely abnormal MRI score from the infants with normal/mild MRI scores, as explained in the MRI scoring system paragraph [6]. Differences between GA, BW, IVH incidence, BPD, NEC, and gender between the normal/mild and moderate/severe MRI WM and GM score groups were checked using the Mann-Whitney and chi-square tests.

Data were analyzed using R program (R Core Team (2016). R: a language and environment for statistical computing. R Foundation for Statistical Computing, Vienna, Austria. URL https://www.r-project.org).

## 3. Results

Clinical characteristics are presented in Table 1. One newborn showed clinical and biochemical signs of mild perinatal asphyxia. None of the infants had an arterial cord blood pH below 7.00. MRIs were performed at a mean postnatal age of 40.9 (SD 0.4) wks. MRI scores are presented in Table 2. None of the patients showed focal cGM abnormalities or dGM volume reduction. No differences in GA, BW, incidence of BPD, or NEC were observed between none/mild and moderate/severe GM and WM MRI scores. The incidence of high-grade IVH significantly was higher in the moderate/severe WM MRI score (*p* < 0.05).

The principal component analysis (PCA) did not show significant clustering between normal/mild abnormal MRI score and moderate/severe MRI abnormalities for all regions (cGM, WM, dGM, and cerebellum) at both time points, 2 days and 10 days after birth. However, ^1^H-NMR urinary metabolic profile analysis showed that lactate and leucine at 2 days were significantly higher in patients with a moderately/severely abnormal WM score compared with infants with a normal or mildly abnormal WM score (see Table 3). Threonine, lactate, and glycine were significantly increased at 2 days in patients with a moderately to severely abnormal cGM score. Acetate signal was significantly increased at 10 days in infants with a moderately/severely abnormal WM MRI score, while carnitine and glycine were significantly higher at the same time point in newborns with a moderately/severely abnormal cGM score.

3-OH butyrate, alanine, and N-acetylated compounds were decreased at 10 days in infants with cGM moderate/severe abnormalities.

**Figure 1:**
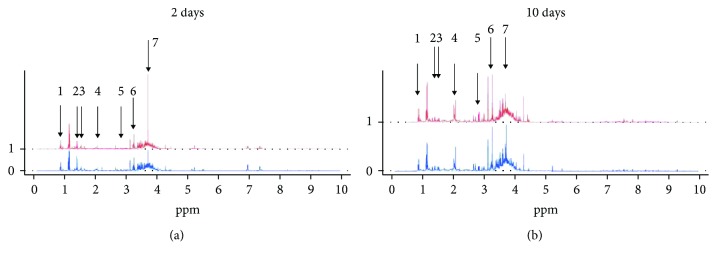
Mean spectra of metabolites in patients with and without a moderately/severely abnormal cGM MRI score (total cGM score ≥ 2) at 2 days (a) and 10 days (b) after birth. Horizontal axis shows ppm. Chemical shifts of relevant signals in ppm: leucine 0.95 and 0.97, threonine 1.33, lactate 1.41, acetate 2.08, citrate 2.89, carnitine 3.22, and glycine 3.72. 0 = normal or mildly abnormal GM score; 1 = moderately-severely abnormal cGM score.

Citrate increased at 10 days in all groups. A complete overview of all the analyzed metabolites is showed in Table 3. An example of spectra for a moderately to severely abnormal cGM score compared to the normal/mildly abnormal group of infants is shown in Figure 1.

**Figure 2:**
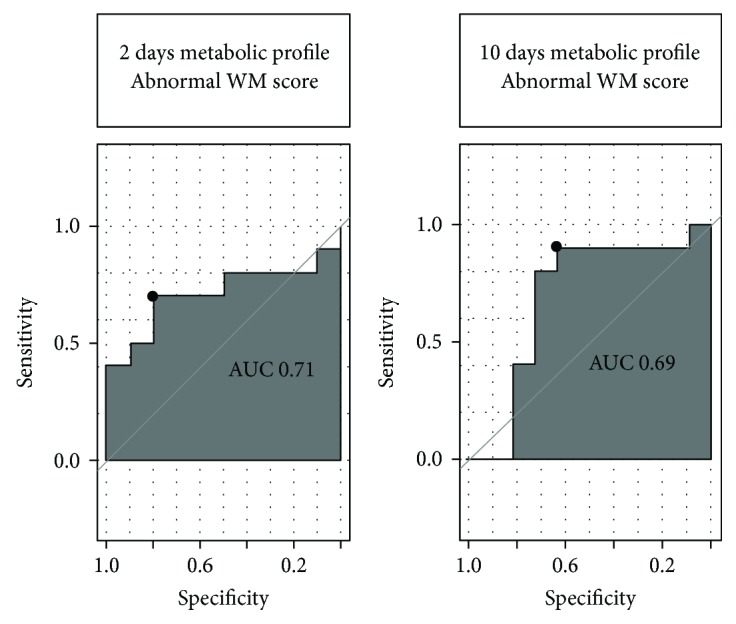
ROC curves showing the predictive value of the panel of metabolites shown in Table 3 for a moderately/severely abnormal WM MRI score (total WM score ≥ 5).

Using the ^1^H-NMR spectra, ROC curves were obtained. The ROC curves for the prediction of moderate-severe WM abnormalities showed the following values: at 2 d, AUC = 0.71, specificity (SP) = 80%, and sensitivity (SE) = 72% and at 10 d, AUC = 0.69, SP = 64%, and SE = 89% (Figure 2).

**Figure 3:**
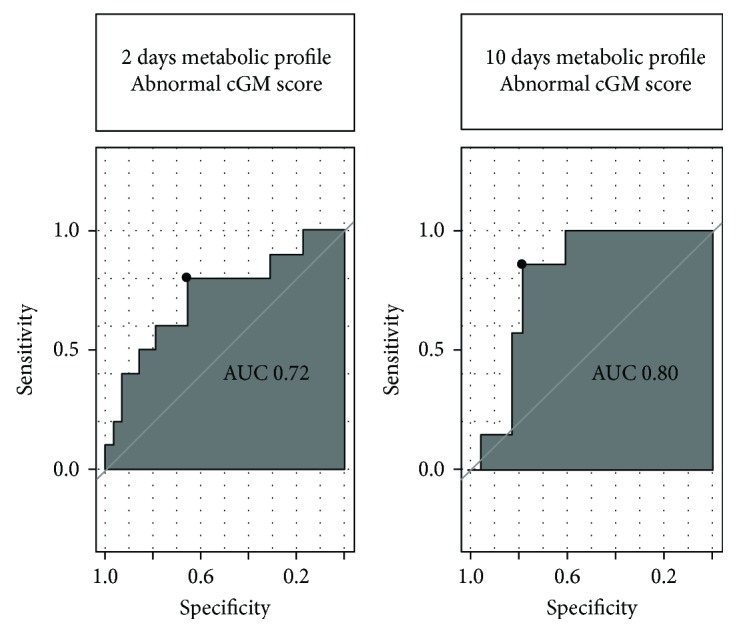
ROC curves showing the predictive value of the panel of metabolites shown in Table 3 for a moderately/severely abnormal cGM MRI score (total cGM score ≥ 2).

The ROC curves distinguished neonates at both 2 and 10 days after birth who later developed a markedly less mature cGM score from the others (with normal or mild abnormalities) (2 d: AUC = 0.72, specificity (SP) = 65%, sensitivity (SE) = 75% and 10 d: AUC = 0.80, SP = 78%, SE = 80%) (Figure 3).

Neither moderately/severely abnormal dGM score nor cerebellum score showed a significant association or could be predicted by the urinary metabolomics profile. Moreover, we could not find any association/prediction between the urinary metabolic profile and moderately to severely abnormal global MRI score.

## 4. Discussion

Specific urinary metabolic spectra of extremely preterm infants at 2 and 10 days after birth were followed by moderately to severely abnormal cGM and WM scores at TEA. This relation was stronger for the cGM score compared to the WM score with a higher specificity and sensitivity. Thus, the present study suggests that, for the first time, metabolomics can provide a valid metabolite signature potentially usable as a prediction model in preterm infants. However, the PCA could not find a significant clustering of metabolic spectra for infants with moderately to severely abnormal MRI scores in all the evaluated regions (WM, cGM, dGM, and cerebellum), since probably the extent of change could depend on many factors and comorbidities such as prematurity-related diseases (ROP, IVH, PDA, length of mechanical ventilation, and sepsis).

Our results suggest that urine is a promising source of biomarkers for brain maturation and that, using metabolomics techniques in urine in the early postnatal period, is useful to identify infants at higher risk of brain disease. Sample collection is easy to do and free from clinical risk. Furthermore, different from blood, urine is always available in preterm newborns.

In our study, several significantly altered metabolites were identified, with different trends over time in infants with normal or mildly abnormal and moderately to severely abnormal MRI scores. These results demonstrate that probably the whole profile is more important for the prediction of the MRI score than the single metabolites.

Analyzing differences in spectra between the normal and mildly abnormal vs. moderately to severely abnormal MRI score, we found that altered metabolites are mainly implicated in the important processes of energy metabolism, mitochondrial function, and myelin production, all impaired in preterm infants [18–21]. In particular, we hypothesize that the increase in lactate and acetate in infants with WM abnormalities is due to the activation of anaerobic glycolysis due to mitochondrial dysfunction and the degeneration of fatty acids, probably due to impaired cerebral blood autoregulation in preterm infants [22]. Developmental immaturity of the preterm brain circulation derives from both altered vasoreactivity and immaturity of vasoactive signaling leads to impaired cerebral blood flow and consequently to intraventricular hemorrhage and ischemic injury to the white matter [23].

Lactate is generally increased after hypoxia, due to the shift from aerobic to anaerobic metabolism [24, 25]. Preterm infants are particularly exposed to cerebral hypoxia, especially in the first days after birth. The main reason is the hemodynamic instability secondary to the transition phase of the respiratory and circulatory systems [26]. Hypoxia may result in an impaired neurodevelopmental outcome in preterm newborns [25].

The white matter is also particularly vulnerable to hypoxic-ischemic and oxidative injury, being sensitive to decreased blood supply [25]. Glycine and threonine increases have been implicated in hypoxia response, oxidative stress, and inflammation, well-known phenomena in preterm infants [24]. Both are glucogenic amino acids, which may be converted to pyruvate during protein metabolism. Increased levels of plasma glycine may be caused by reduced amino acid oxidation or reduced gluconeogenesis as a strategy to conserve amino acids [27]. In most mammals, the pre-postnatal transition is accompanied by important adaptations in carbohydrate metabolism due to the abrupt change from the placental supply of nutrients to a cyclic supply via breast milk; however, preterm infants experience enteral feeding difficulties in the neonatal period, linked to the impaired gut function [27]. Furthermore, glycine is a necessary substrate for the biosynthesis of reduced glutathione, the main antioxidant molecule in the brain [28].

Although we believe that urine metabolomics represents a promising noninvasive approach to study brain diseases in the neonatal population, data are too limited to draw definite conclusions regarding the use of metabolomics profile in clinical practice. Potential confounders should be analyzed in detail and will benefit from studies on a larger number of patients to identify the effect of environmental factors and comorbidities on the metabolomics spectra. A validation of our results in a new and larger cohort of extremely preterm infants is also necessary to check reproducibility of spectra. Furthermore, the effect of nutrition might have played an essential role, although all infants received the same parenteral nutrition solution and the increase in oral feeding was performed using the same steps, following the local protocol of the UMC Utrecht. Finally, the lack of standards in both analytical methods as well as “normality ranges” for urine metabolites in newborns is a big limitation to this promising technique [29].

## 5. Conclusions

Urine spectra of extremely preterm infants at 2 and 10 d discriminate patients with a moderately to severely abnormal cGM score and WM score at TEA. The main metabolic changes could be connected to 2 different metabolic pathways: energy metabolism and protein metabolism. Thus, urine ^1^H-NMR appears to be a promising tool for early identification of neonates at high risk of subnormal brain development and for better understanding of the multifactorial pathogenesis of neonatal brain injury [30]. However, studies on a larger number of patients are needed to confirm our findings.

## Figures and Tables

**Table 1:** Clinical characteristics of the population.

Baseline characteristics	*N* total = 30
GA (wks), mean (SD)	26.6 (1.0)
BW (gr), mean (SD)	911 (178)
Gender, male (%)	15 (50)
Apgar score 1st min, median (IQR)	5 (3–7)
Apgar score 5st min, median (IQR)	8 (7–8)
Arterial umbilical cord pH, mean (SD)	7.24 (0.09)
Arterial umbilical cord BE mmol/l, mean (SD)	−5.1 (4.0)
Arterial umbilical cord lactate mmol/l, mean (SD)	5.0 (2.9)
PDA surgery, *n* (%)	2 (6.7)
BPD, *n* (%)	7 (23.3)
Late-onset sepsis, *n* (%)	4 (13.3)
IUGR, *n* (%)	2 (6.7)
IVH, *n* (%)	
Grades 1-2	6 (20)
Grades 3-4	3 (10)

Abbreviations: GA: gestational age: BW: birth weight; SD: standard deviation; BE: base excess; PDA: patent ductus arteriosus; BPD: bronchopulmonary dysplasia; IVH: intraventricular hemorrhage; IR: interquartile range.

**Table 2:** MRI classes, using the Kidokoro MRI score, in the study group. Abbreviations: WM: white matter; cGM: cortical grey matter; DGM: deep gray matter; PLIC: posterior limb of the internal capsule; CR: corona radiata; IHD: interhemispheric distance; cTCD: coronal transverse-cerebellar diameter.

MRI subscores	*N* = 30	MRI total sores	*N* = 30
*MRI WM score subitems*		*MRI WM score classes*	
Cystic lesions *n* (%)	4 (13.3)	Normal	4 (13.3)
Focal unilateral	1 (3.3)	Mild abnormalities	16 (53.4)
Extensive unilateral	3 (10)	Moderate	4 (13.3)
Myelination		Severe	6 (20)
Normal PLIC for TEA	27 (90)		
Ventricular dilatation			
Both sides < 7.5 mm	22 (73.4)		
One side 7.5–10 mm	4 (13.3)		
Both sides 7.5–10 or 1 > 10	Side 4 (13.3)		
*cGM score subitems*		*cGM score classes*	
Signal abnormalities	0 (0)	Normal	8 (26.6)
Gyral maturation		Mild abnormalities	10 (33.3)
Delay <2 wks	11 (36.7)	Moderate	5 (16.7)
2–4 wks	18 (60)	Severe	7 (23.3)
>4 wks	1 (3.3)		
Increased IHD			
IHD < 4 mm	17 (56.6)		
4–5 mm	6 (20)		
–6 mm	2 (6.7)		
>6 mm	5 (16.7)		
*DGM score subitems*		*DGM score classes*	
Signal abnormalities		Normal	28 (93.4)
Focal unilateral	1 (3.3)	Mild abnormalities	1 (3.3)
Focal bilateral	1 (3.3)	Moderate	1 (3.3)
Volume reduction	0 (0)	Severe	0 (0)
*Cerebellum score subitems*		*Cerebellum classes*	
Punctate unilateral	6 (20)	Normal	3 (10)
Punctate bilateral	1 (3.3)	Mild abnormalities	19 (63.3)
Volume reduction		Moderate	6 (20)
cTCD > 50 mm	24 (80)	Severe	2 (6.7)
47–50 mm	5 (16.7)		
47	1 (3.3)		
*Global MRI classes*			
Normal	3 (10)		
Mild abnormal	19 (63.3)		
Severe	2 (6.7)		

**Table 3:** Summary of altered metabolites extract from PCA analysis of urine average ^1^H-NMR spectra. The circles and stars indicate the score of increasing metabolites compared to the average spectra (from low (1 circle) to very high (>10 circles)). Stars: significant metabolites. Abbreviations: 3-OHbut: 3-hydroxybutyrate; 3-NH_2_isobut: 3-aminoisobutyrate; 3-OHisoval: 3-hydroxyisovalerate; N-acethylated: N-acethylated compounds; dma: dimethylamine; dimethylgly: dimethylglycine; NMNA: N-methylnicotinamide.

Metabolites	2 days				10 days			
	WM score		cGM score		WM score		cGM score	
	Normal	Moderate	Normal	Moderate	Normal	Moderate	Normal	Moderate
	Mild	Severe	Mild	Severe	Mild	Severe	Mild	Severe
	*N* = 20	*N* = 10	*N* = 18	*N* = 12	*N* = 20	*N* = 10	*N* = 18	*N* = 12
Leucine	∗	∗∗∗			°	°	°	°
3-OHbut							∗∗∗	∗∗
3-NH_2_isobut			°°	°				
3-OHisoval	°	°			°°	°°	°	°
Threonine			∗	∗∗∗	°	°	°°	°°
Lactate	∗∗	∗∗∗∗	∗	∗∗				
Alanine	°		°°°°	°°°°	°°°°	°°°°	∗∗∗∗∗∗	∗∗
N-Acethylated at 2.02 ppm					°°°°°°	°°°°°°	∗∗∗∗∗∗∗∗∗∗∗∗∗	∗∗∗∗∗∗∗∗
N-Acethylated at 2.05 ppm					°°	°°	°°°°°°	°°
Acetate	°	°				∗∗	°	°
Acetone	°°	°°	°	°				
Pyruvate							°	°
Succinate	°	°	°°	°°	°°	°°	°°	°°
dma			°	°	°°°°	°°°°		
Dimethylglycine	∗∗∗∗	∗∗			°	°	°	°
Citrate	∗∗∗∗	°°°°	∗∗	°°	∗∗∗∗∗∗∗∗∗∗	°°°°°°°°°°°	∗∗∗∗∗∗∗	°°°°°°°
Creatine	°	°			°	°	°	°
Creatinine	°°°°	°°°°	°°°°	°°°°	°°°°°°°°°°	°°°°°°°°°°	°°°°°°°	°°°°°°°°
Carnitine	°°°	°°°	°°°	°°°	°°°	°°°	∗∗	∗∗∗∗
Betaine	°°°°°°	°°°°°°	°°°°°	°°°°°	°°°°°°	°°°°°°	°°°°°°	°°°°°°
Myoinositol	°°	°°	°°°°	°°°	°°°°°°°	°°°°°°°°	°°	°°
Glycine	°°°°°	°°°°°	∗	∗∗∗	°°°°	°°°°	∗∗∗	∗∗∗∗
Glucose	°°°°°	°°°°°	°°°°°°	°°°°°°	°°°°	°°°°	°°°°°°°	°°°°°°°
Fumarate							°	°
Hippurate					°°°°°°°°°	°°°°°°°°°	°°	°°
Formiate	°°	°°	°°°°°	°°°	°°°	°°°	°	°
NMNA			°°	°°	°	°	°	°

## Data Availability

The dataset and urine samples used to support the findings of this study are available from the corresponding author upon request.

## Abbreviations

^1^H-NMR: Proton magnetic resonance spectroscopy
AUC: Area under the curve
BPD: Bronchopulmonary dysplasia
BW: Birth weight
cGM: Cortical grey matter
dGM: Deep grey matter
GA: Gestational age
IVH: Intraventricular hemorrhage
MRI: Magnetic resonance imaging
NEC: Necrotizing enterocolitis
NICU: Neonatal intensive care units
ROC: Receiver-operating characteristic
ROP: Retinopathy of prematurity
PCA: Principal component analysis
PDA: Patent ductus arteriosus
PMA: Postmenstrual age
PLSDA: Partial least square discriminant analysis
SE: Sensitivity
SP: Specificity
TEA: Term equivalent age.
